# Developing a carbon footprint calculation method for product life cycle based on low-carbon design: A case study of the STAGE Bluetooth speaker

**DOI:** 10.1371/journal.pone.0327576

**Published:** 2025-08-20

**Authors:** Yuwei Zhang, Shanliang Yao, Yantao Chen

**Affiliations:** 1 School of Art and Design, Wuhan Institute of Technology, Wuhan, China; 2 Department of Mechanical Engineering, Faculty of Engineering, Universiti Malaya, Kuala Lumpur, Malaysia; 3 Ningbo Yuandao Industrial Design Co., Ningbo, China; Manipal Academy of Higher Education, INDIA

## Abstract

In the field of low-carbon design, the collection and quantitative analysis of carbon footprint data are vital for reducing the environmental impact of products. This paper proposes a carbon footprint calculation method based on the product life cycle, using Cleer’s STAGE Bluetooth speaker as a case study. By integrating greenhouse gas (GHG) emission data from the GaBi database with the life cycle assessment (LCA) theory and the PAS 2050 standard, a carbon emission quantification model and calculation method for Bluetooth speakers have been developed. Results indicate that the STAGE Bluetooth speaker has a total carbon footprint of 227.01 kgCO₂e across its life cycle. The use stage contributes the most (72.77%, 165.2 kgCO₂e), followed by transportation (25.23%, 57.28 kgCO₂e), raw material acquisition (3.78%, 8.6 kgCO₂e), and production (1.82%, 4.13 kgCO₂e). The recycling of materials results in a net reduction of −3.6% (−8.2 kgCO₂e). The research demonstrates that improving energy efficiency and substituting materials are crucial strategies for reducing the carbon footprint of electronic products. This method offers a theoretical basis for low-carbon design in similar products and serves as a reference for assessing the carbon footprint of other electronic devices in industrial applications.

## Introduction

Climate change is one of the most severe global issues in recent years [1]. According to the World Meteorological Organization (WMO), the current global temperature has risen by nearly 1°C compared to pre-industrial levels, with projections indicating a 3–5°C increase by 2100 [2]. Against this backdrop, the transition to a green low-carbon economy has become a global priority, driving the need for precise carbon footprint assessment methodologies as a core technical pathway to achieve “dual-carbon” goals [3]. Life cycle assessment (LCA), as the cornerstone of carbon accounting, has evolved significantly in multidisciplinary research. For instance, hybrid LCA techniques integrating process analysis and input-output methods have enhanced the accuracy of carbon footprint evaluations for electronic products [4]. However, such methods face practical challenges due to their reliance on transparent supply chain data, especially in industries with fragmented data systems [4]. Similarly, the functionality-based LCA framework introduces dynamic user behavior considerations into carbon emission analysis for consumer electronics [5], yet its application is hindered by ambiguities in functional boundaries, leading to significant uncertainties. Cordella (2021) demonstrates the emission reduction potential of lifespan extension, modular design, and material recycling in smartphones [6], but their applicability to audio devices remains unverified. While modular upgradability in consumer electronics offers carbon reduction through component replacement [7], it lacks quantitative analysis of the relationship between design features and carbon footprints. Despite methodological progress, critical limitations persist in existing research, including incomplete system boundaries, where most studies truncate at the use stage, neglecting emission disparities in recycling treatments, resulting in systematic underestimation [8]. At the same time, in typical residential communities in China, household energy consumption carbon emissions account for more than 50% of total household emissions [9], with household appliances being the main driving factor. Therefore, these appliances also generate considerable carbon emissions during production, transportation, and disposal.

To address these research gaps, this study proposes a method for calculating the carbon footprint of Bluetooth speakers over their entire life cycle based on LCA theory and the PAS 2050 standard [10]. Quantifying the carbon emissions of speakers at each stage, this method reveals the low-carbon optimization space and the correlation between emissions across stages [5]. The innovation is reflected in three aspects: (1) constructing a carbon accounting model applicable to enterprises that integrates enterprise monitoring data and GaBi regional parameters; (2) verifying the reliability of the traditional 1g mass threshold and proposing mandatory inclusion criteria for trace materials with high emission factors (EFs) [11]; (3) identifying key emission reduction paths for energy efficiency improvement and material substitution. The research results provide a theoretical basis for the low-carbon design of speakers, a reference for the optimization of similar home appliances, and establish a methodological basis for the carbon footprint assessment of other electronic products.

This study is divided into five parts: the first part systematically analyzes the application of relevant methods through literature synthesis, and critically reviews existing research to identify methodological gaps and propose improved solutions; the second part describes the carbon footprint calculation method of products; the third part verifies the method through a case data study of the STAGE Bluetooth speaker; the fourth part conducts an in-depth discussion of the research results; the fifth part summarizes the research and proposes directions for future research.

## Materials and methods

To calculate the carbon footprint, we initially employ the life cycle approach guided by PAS 2050. After setting the calculation objectives aligned with the standard’s requirements, we construct a flowchart of the product’s life cycle covering raw material acquisition, production, transportation, use, and recycling stages [12]. System boundaries are rigorously defined following PAS 2050’s business-to-consumer (B2C) model to ensure full life cycle coverage. Within these boundaries, enterprise-level raw data, including production energy consumption, transportation logs, and usage patterns, were obtained through a donation agreement signed with the research team from Shenzhen Guanxu Electronics Co., Ltd., a leading consumer electronics manufacturer. Anonymized operation records and field monitoring data were provided under authorized access and supplemented with region-specific EFs and pre-modeled industrial processes from the GaBi database [13]. This study adopts the following principles to ensure calculation accuracy: (1) Component screening rules: Ignore components with a mass less than 1g but retain materials with high EFs. Components exceeding the threshold are included in the calculation. (2) Data integration process: Enterprise-level data is obtained through production line monitoring and cross-validated with GaBi regional parameters to ensure data representativeness and regional adaptability. Finally, the collected data are input into the calculation framework, where PAS 2050’s standardized formulas for transportation emissions and LCA tools jointly quantify emissions, identify high-impact stages, and inform low-carbon optimization strategies.

### PAS 2050 evaluation criteria

The applicability of the PAS 2050 standard in this study stems from its targeted methodological support for consumer electronics products. The standard was developed by the British Standards Institution (BSI), and its business-to-business (B2B) and business-to-consumer (B2C) models accurately divide the accounting boundaries between the component level and the finished product level. This division fully aligns with the full life cycle model of this study, covering “raw materials—production—transportation—use—recycling”. PAS 2050 not only covers six types of greenhouse gases (GHGs) such as CO₂ and CH₄ but also allows for the dynamic integration of regionalized parameters and the mandatory inclusion of materials with high EFs, effectively addressing the data fragmentation problem of enterprises [14]. This feature directly supports the core innovation of this study: by integrating the GaBi global database and regionalized parameters, a calculation model that considers both scientific rigor and practical feasibility is constructed to provide a decision-making framework for the low-carbon design of consumer electronic products [15].

### STAGE Bluetooth speaker overview

**Fig 1:**
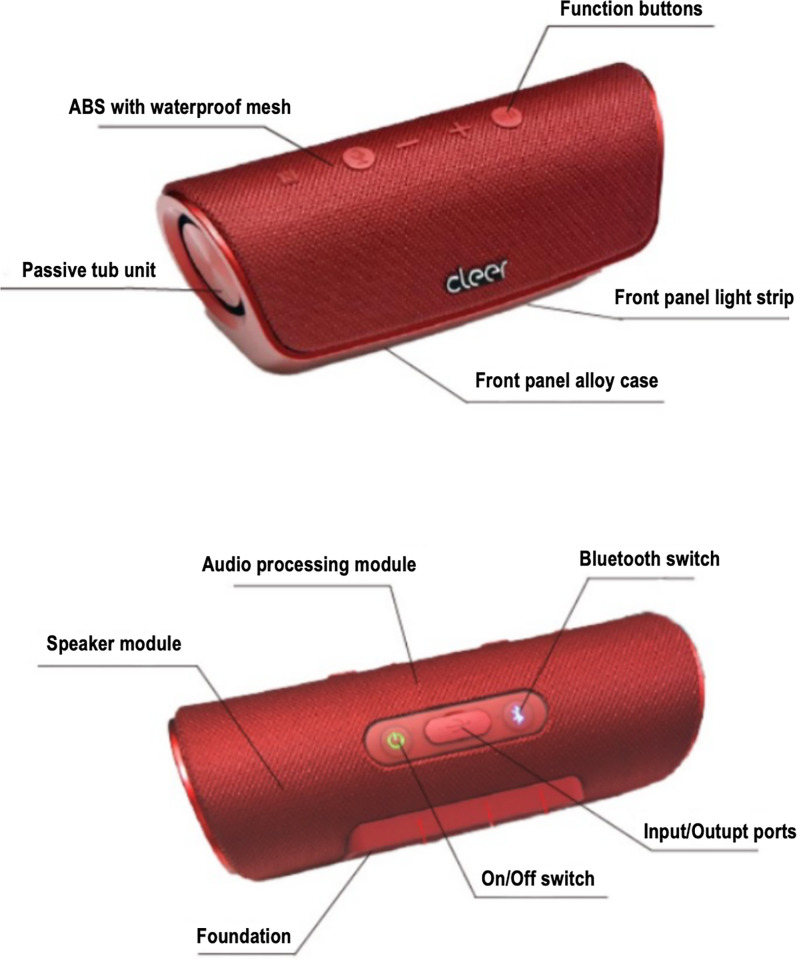
STAGE bluetooth speaker diagram.

STAGE is a portable Bluetooth speaker under the Cleer acoustic brand, holding a significant market share in the Bluetooth speaker industry and widely popular among consumers. Therefore, it can effectively represent the current development level of the Bluetooth speaker industry. This product has been chosen as the research subject of this paper. The following figure is a schematic diagram of the product. See Fig 1.

Based on the disassembly and functional analysis of product components, the Bluetooth speaker is divided into four functional modules: the shell and bracket system, the electronic components system, the power supply system, and the packaging system. The main component information for each module is shown in the table below. See Table 1.

**Table 1:** STAGE Bluetooth Speaker Parts Information.

Pseudolaric acid		Cleer Bluetooth Speaker
Product Model		STAGE
**Structural description**		
**System**	**Component Name**	**Detailed Description**
**Shell and Bracket System**	Main Frame	The inner structure frame of the speaker
	Decorative Strip	ABS material decorative strip, installed outside the frame, serves to cover and protect the interior and contributes to the overall aesthetic
	Bottom Non-slip Pad	Increases the stability of the speaker placement
	Speaker Net	Speaker net component is composed of ABS frames with attached fabric on the outside; functions to protect the speaker and prevent dust
	Metal Radiating Diaphragm Unit	Oscillates with the rhythm of the music
**Electronic Component System**	MAIN Mainboard Component	Main control board
	USB & AUX Board Component	Output/input unit
	ON & OFF Board Component	Switch
	LED Board Component	LED lights
	KEY Board Component	Development board
	Speaker L48	Speaker unit
**Power Supply System**	Battery Group	Cylindrical lithium-ion battery (7.4V, 2600mAh)
**Packaging System**	User manual, EVA shockproof, color box, inner support, outer box, stickers	Packaging for logistics and product sales

### STAGE Bluetooth speaker carbon emission quantification model

**Fig 2:**
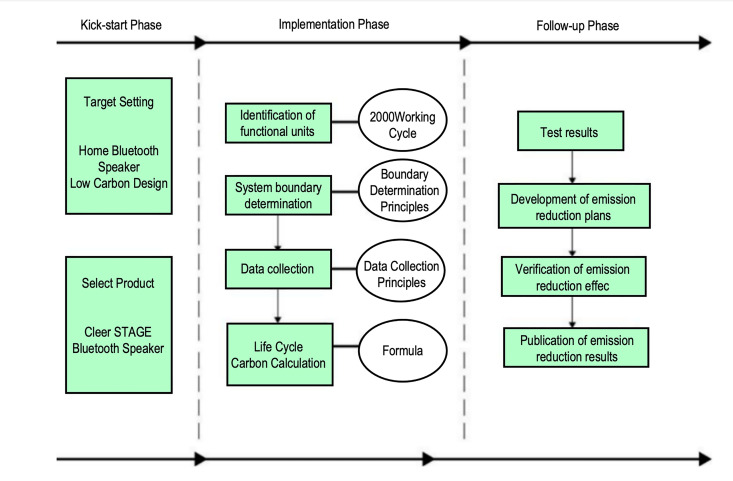
STAGE bluetooth speaker carbon emission quantitative assessment model.

Based on the theory of LCA and the PAS 2050 assessment standard, a carbon emission assessment model for Bluetooth speakers has been established, with the functional unit defined as 2500 working cycles [16]. This model encompasses three stages: goal definition, data collection, and calculation. The specific procedure for carbon emission assessment is as follows: First, determine the system boundaries of the product; then, collect the data required for carbon emission calculations; finally, apply the calculation formulas using the collected data, thereby determining the carbon emissions of the Bluetooth speaker during the main stages of its life cycle [17]. See Fig 2.

### STAGE Bluetooth speaker carbon footprint system boundaries established

In environmental assessment and life cycle analysis, the definition of system boundaries is a crucial step that determines the scope of life cycle stages covered in product data collection. System boundaries are explicit specifications of the methods, scope, and depth of product data collection, ensuring that all key stages in the product life cycle are included. This not only aids in accurately measuring the product’s environmental impact but also helps identify potential opportunities to improve efficiency and reduce impact [18].

In the assessment of product carbon footprints, there is a difference in setting system boundaries between components and the final product. For components, the system boundary for carbon footprint assessment typically adopts a B2B model, which only considers the carbon emissions during the transfer of components from one enterprise to another. In contrast, for the final product, the assessment system boundary adopts a B2C full life cycle model, calculating the entire life cycle carbon emissions from raw material acquisition and production to the product being used by consumers and its disposal [19]. In the selection of temporal boundaries, studies often choose a full year, a specific period, or the entire cycle from production to sales of a particular batch of products as the temporal boundary for carbon accounting, to eliminate the impact of seasonal fluctuations and facilitate comparison between carbon footprint results of different periods or batches of products [20].

**Fig 3:**
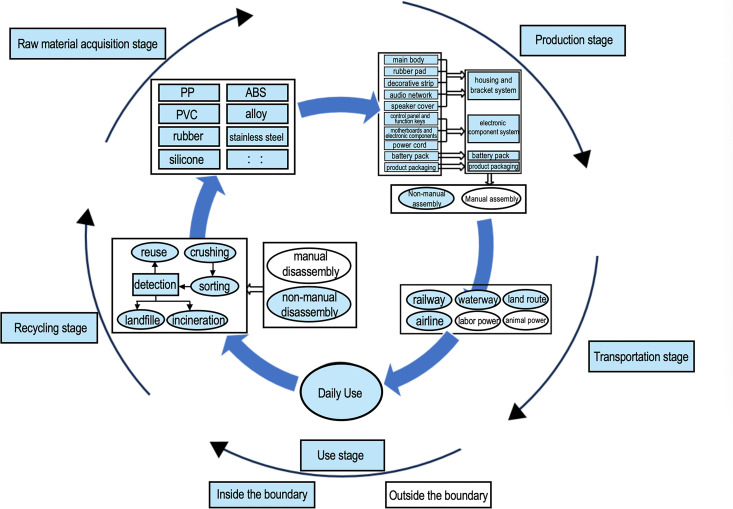
STAGE bluetooth speaker carbon footprint calculation system boundary.

In the disassembly and testing of the STAGE Bluetooth speaker sample, the acquisition of experimental data and its feasibility greatly determine the setting of system boundaries. In this study, based on the life cycle analysis method of PAS 2050, the system boundaries for Bluetooth speakers are defined using the B2C model [21]. The assessment scope includes the carbon emissions throughout the entire life cycle of the product: raw material acquisition and transportation, product assembly, sales transportation, use, and recycling treatment [10]. See Fig 3.

### Data collection methods within boundary

In conducting a carbon footprint assessment, based on the established assessment boundaries, it is necessary to collect two types of key data for calculating carbon emissions:

The first category is activity data, which quantifies the intensity or magnitude of operations within each process unit, such as production quantity, transportation distance, energy consumption, etc. Activity data can be obtained through on-site monitoring of the production process to get primary data. Alternatively, secondary data sources such as a company’s production and operational records can also be referenced [22].

The second category is EF data. EFs reflect the amount of GHG emissions corresponding to a unit of activity [23].

When collecting activity data from primary monitoring, it is important to control the impact of random measurement errors on data quality. Errors can be minimized through repeated monitoring and equipment calibration, ensuring that data quality remains within acceptable thresholds. At the same time, the representativeness of the monitoring data must be considered to avoid insufficient sample sizes that fail to reflect the overall activity level.

To comprehensively assess the carbon emission impact of a product on the environment, the product carbon footprint accounting should follow the principles of LCA, examining the carbon emissions throughout the entire life cycle of the product [24]. Specifically, the full life cycle carbon accounting should include the following stages [25]:

(1) Raw material acquisition stage. In this stage, the energy consumed and the corresponding GHG emissions in obtaining various raw materials need to be accounted for [17].
(2) Production and manufacturing stage. During the factory production of the product, the energy consumption and carbon emissions from the treatment and disposal of various waste materials must be considered.
(3) Use stage. The energy consumption of the product when it is acquired and used by consumers should be included in the carbon accounting scope.
(4) Transportation stage. The carbon emissions caused by the transportation of raw materials to production factories and the delivery of products from factories to consumers must be accounted for [26].
(5) Recycling and disposal stage. The carbon emissions are associated with the recycling, reuse, and final disposal of the product after it is scrapped.

Only by accounting for the GHG emissions of each stage within the entire life cycle of the product can we scientifically and comprehensively assess the carbon footprint of the product and its environmental impact [26]. This is the basic method and requirement for conducting product carbon accounting [12].

To further quantify the parameter uncertainty in LCA, this study introduced the Monte Carlo simulation method to support the sensitivity analysis of carbon footprint results. Key uncertainty parameters, including component mass, regional EFs, energy consumption during the use stage, and material recycling efficiency, were assigned probabilistic distributions based on actual production data and literature support, enabling a probabilistic evaluation of the 1g mass threshold and emission reduction strategies [27].

### STAGE Bluetooth speaker life cycle carbon footprint calculation and formulation

Based on the theory of LCA and employing the PAS 2050 guidelines, the carbon emission calculation formulas for each stage of the STAGE Bluetooth speaker’s life cycle, as well as the total carbon emissions throughout the life cycle process [28], have been established as shown in Table 2:

**Table 2:** Calculation formula for the carbon footprint of STAGE product life cycle.

Life cycle stage	Formula	Formula Explanation
Raw material acquisition stage	$\text{Gm}=\sum_{i=1}^{m}\left(M_{i}\times EF_{i}\right)$	1、Gm: GHG emissions at raw material acquisition (kgCO₂e). 2、${\text{M}}_{\text{i}}$: Mass of material i (kg) 3、$EF_{i}$: Emission factor of material i (kgCO₂e/kg).
Production and manufacturing stage	$\text{Gp}=\sum_{i=1}^{n}\left(E_{i}\times EF_{i2}\right)$	1、Gp: GHG emissions from production energy (kgCO₂e). 2、$E_{i}$: Energy consumption of process i (kWh). 3、$EF_{i\text{2}}$: Emission factor of energy source i\ (kgCO₂e/kWh).
Transportation stage	$\text{Gt}=\sum_{i=1}^{n}\left(T_{i}\times D_{i}\times EF_{i3}\right)$	1、Gt: GHG emissions from transportation (kgCO₂e). 2、$T_{i}$: Transported weight of category i (kg). 3、${\text{D}}_{\text{i}}$: Transportation distance for category i (km). 4、$EF_{i\text{3}}$: Emission factor of transport mode i (kgCO₂e/km·kg).
Use stage	$\text{Gu}=E\times T_{w}\times EF_{i34}$	1、Gu: GHG emissions from product use (kgCO₂e). 2、E: Power consumption per cycle (kWh). 3、${\text{T}}_{\text{w}}$: Total working cycles. 4、$EF_{i\text{34}}$: Grid electricity emission factor (kgCO₂e/kWh).
Recycling and disposal stage	$\text{Gr}=\sum_{i=1}^{n}\left(E_{ir}\times EF_{i5}\right)-\sum_{j=1}^{n}\left(M_{jr}\times EF_{jr}\right)$	1、Gr: Net GHG emissions from recycling (kgCO₂e). 2、$E_{ir}\text{\textbackslash{} }$: Energy consumed in recycling process i (kWh). 3、$EF_{i\text{5}}$: Emission factor of recycling energy i (kgCO₂e/kWh). 4、$M_{jr}$: Mass of recycled material *j* (kg). 5、$EF_{jr}$: Avoided emission factor for material *j* (kgCO₂e/kg).

The GHG emissions during the raw material acquisition stage (Gm) are calculated by multiplying the mass of each material (${\text{M}}_{\text{i}}$) by its corresponding emission factor ($\text{E}{\text{F}}_{\text{i}}$). The production and manufacturing stage (Gp) accounts for energy consumption data, while the transportation stage (Gt) integrates transportation weight (${\text{T}}_{\text{i}}$), distance (${\text{D}}_{\text{i}}$), and mode-specific emission factors ($\text{E}{\text{F}}_{\text{i3}}$). The use stage (Gu) and recycling and disposal stage (Gr) are similarly modeled with their respective parameters.

## Results

In this study, carbon emission data for the STAGE Bluetooth speaker’s life cycle were systematically collected through factory experiments, supply chain audits, and laboratory testing. Key parameters, including material masses, energy consumption, transportation logs, and usage patterns, were sourced from enterprise records and cross-validated with the GaBi database. Components below the 1g mass threshold were excluded after the sensitivity analysis.

### STAGE Bluetooth speaker life cycle data collection

This study collected carbon emission data for each stage of the STAGE Bluetooth speaker’s life cycle, including the data required to calculate the carbon emissions at each corresponding stage [29]:

(1) Raw material acquisition stage

The raw material acquisition stage mainly considers the direct and indirect GHG emissions caused by the energy consumption for producing materials such as steel, iron, copper, glass fiber, and plastic. By summarizing and organizing the bill of materials of the STAGE Bluetooth speaker, these materials are classified into five categories: plastic parts, silicone parts, hardware parts, electrical components, and others [30].

The names, quantities, and masses of the components required for the STAGE Bluetooth speaker are provided by Cleer’s component procurement department. After obtaining a summary list of the product’s parts and materials, based on the criteria for data selection [18], GHG emissions from parts with a mass less than 1g are disregarded. The mass of disregarded parts accounts for 0.25% of the total product mass, which is less than 5% of the product’s weight. See Table 3.

**Table 3:** STAGE Bluetooth Speaker Bill of Materials and Carbon Emission Factors.

Material type	Component Name	Material	Quantity	Total Weight (g)	Carbon Emission Factor (kgCO₂e)	Carbon Footprint (kgCO₂e)
**plastic part**	Main Frame	ABS	1	142.58	16.6	2.367
	Function Key Cover	ABS	1	4.6	16.6	0.076
	Diaphragm Bracket	ABS	2	34.2	16.6	0.57
	Main Foot Stand	ABS	1	13.5	16.6	0.22
	LED Back Cover	ABS	1	5.28	16.6	0.088
	Decorative Strip	PC	1	31.6	0.2	0.006
	Function Key Top Cover	PC	1	4.1	0.2	0.0008
	USB Cover	TPU	1	1.25	4.25	0.018
	TYPEC Input Box Top Cover	TPU	1	4.65	4.25	0.02
	Speaker Grid Component	ABS Component/Waterproof Cloth	1	90.5	16.6	1.5
**Silicone parts**	Main Bracket Rubber	Rubber	1	17.7	2.4	0.29
	Sealing Ring	Rubber	2	4.7	2.4	0.0113
**Hardware**	Screw	Carbon Steel	58	11.88	3.2	0.038
	Passive Basin	Aluminum	2	61.2	1.8	0.11
**electronic component**	PVC Line	PVC	2	3.6	7	0.025
	MAIN Mainboard Component (Integrated Chip)	Component	1	41.5	29.33	1.22
	USB & AUX Board Component	Component	1	1.95	29.33	0.06
	ON & OFF Board Component	Component	1	1.93	29.33	0.06
	LED Board Component	Component	1	2	29.33	0.059
	KEY Board Component	Component	1	3.06	29.33	0.09
	Speaker L48	Component	2	140	29.33	0.533
**Other**	Battery Group	Component	1	99	6.31	0.624
	Sticker	Copper Plate Paper	11	1.1	3	0.0033
	Color Box (including inner holder)	Gray Card Paper	1	403	1.7	0.68
	Instruction Manual	Copper Plate Paper	1	6.8	3	0.0204
	Shockproof EVA	EVA	16	4.6	2.82	0.013

(2) Production and manufacturing stage

The production and manufacturing stage primarily considers the direct GHG emissions and electricity consumption associated with the manufacturing of major components, motherboard burn-in and testing, installation of electrical components, installation of fixings and speaker units, various other tests, conveyor belt transportation, and the interconnections between different processes.

**Fig 4:**
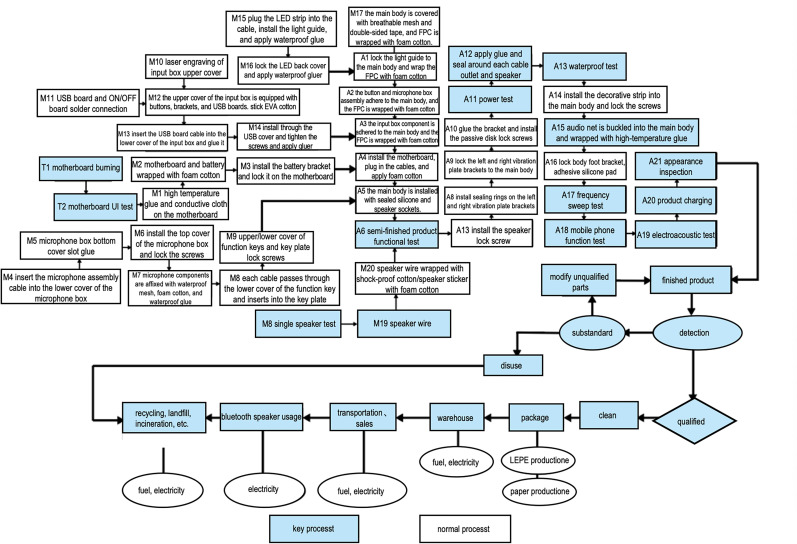
STAGE bluetooth speaker production process flow chart.

This paper presents the main production process flow of the Bluetooth speaker, obtained through on-site enterprise research, as shown in Fig 4.

The data list for each manufacturing system is provided in Table 4. Most of the processes in the workflow are labor-intensive, with a total electricity consumption of 10 kWh during the production stage. The carbon footprint for this period was calculated based on the EF of the Southern Power Grid and local enterprise data and adjusted to 0.413 kgCO₂e/kWh [31].

**Table 4:** Energy consumption of STAGE bluetooth speaker during production.

#	Manufacturing Step	Labor	Energy Source (Electricity/Steam/Natural Gas/Oil/Biofuel, etc.)
**T1**	Mainboard Programming	1	Electricity
**T2**	Mainboard UI Testing	1	Electricity
**M1**	Mainboard High-temperature Glue and Conductive Cloth Application	1	Electricity
**M2**	Mainboard and Battery Foam Wrapping	1	Manual Work
**M3**	Battery Bracket Installation and Fastening on Mainboard	1	Manual Work
**M4**	Microphone Component Assembly and Installation into Microphone Box Lower Cover	1	Manual Work
**M5**	Gluing of Microphone Box Lower Cover Slot	1	Manual Work
**M6**	Upper Cover Installation and Screw Fixation for Microphone Box	1	Electricity
**M7**	Microphone Component Waterproofing and Foam Installation	1	Manual Work
**M8**	Function Key Cable Routing and Plug into Key Panel	1	Manual Work
**M9**	Function Key Upper/Lower Cover and Button Panel Screw Fixation	1	Electricity
**M10**	Input `Box Upper Cover Laser Engraving	1	Electricity
**M11**	USB Board and ON/OFF Board Soldering	1	Electricity
**M12**	Input Box Upper Cover Assembly: Key Bracket Installation and USB Board Foam Sticking	1	Manual Work
**M13**	USB Board Cable Routing, Installation into Input Box Lower Cover and Gluing	1	Manual Work
**M14**	USB Cover Installation into Upper Cover, Screw Fixation and Gluing	1	Manual Work
**M15**	LED Panel Cable Assembly, Installation into Light Guide and Waterproofing	1	Manual Work
**M16**	LED Back Cover Screw Fixation and Waterproofing	1	Manual Work
**M17**	Main Body Air Vents and Double-sided Tape Application, Microphone FPC Foam Wrapping	1	Manual Work
**M18**	Single Speaker Testing	1	Electricity
**M19**	Speaker Wire Soldering	1	Electricity
**M20**	Speaker Wire Vibration Cushion and Speaker Foam Application	1	Manual Work
**A1**	Light Guide Installation onto Main Body, FPC Foam Wrapping	1	Manual Work
**A2**	Button Component and Microphone Component Adhesion onto Main Body, FPC Foam Wrapping	1	Manual Work
**A3**	Input Box Component Adhesion onto Main Body, FPC Foam Wrapping	1	Manual Work
**A4**	Mainboard Installation, Cable Plugging, Foam Application	1	Manual Work
**A5**	Main Body Silicone Seal Installation, Speaker Socket Assembly	1	Electricity
**A6**	Semi-finished Product Functional Testing	1	Electricity
**A7**	Speaker Installation and Screw Fixation	1	Electricity
**A8**	Left and Right Vibration Plate Bracket Seal Ring Installation	1	Manual Work
**A9**	Left and Right Vibration Plate Bracket Installation onto Main Body	1	Manual Work
**A10**	Bracket Gluing, Passive Disc Screw Fixation	1	Electricity
**A11**	Power Testing	1	Electricity
**A12**	All Cable Port and Speaker Area Sealing	1	Manual Work
**A13**	Waterproof Testing	1	Electricity
**A14**	Decorative Strip Installation into Main Body and Screw Fixation	1	Electricity
**A15**	Speaker Mesh Fastening into Main Body and High-temperature Tape Wrapping	1	Electricity
**A16**	Sweep Frequency Testing	1	Electricity
**A17**	Phone Function Testing	1	Electricity
**A18**	Electroacoustic Testing	1	Electricity
**A19**	Product Charging	1	Electricity
**A20**	Appearance Check	1	Manual Work

(3) Transportation stage

The transportation stage is divided into the supply process from component manufacturers to the producing enterprises, the distribution process from producing enterprises to product sales enterprises, the process of consumers purchasing from product sales enterprises, and the process of waste transportation to recycling enterprises. Considering that during the purchasing process, there are no significant transportation stages, and the importance of this stage is far less than that of other life cycle stages, the principle of omission has been applied to discard this stage. Based on data obtained from factory experiments, the data list for the main transportation stages of the Bluetooth speaker is provided in Table 5.

**Table 5:** Data list of main transportation stages of STAGE bluetooth speaker.

Item	Transportation Weight	Origin	Destination	Mode	Distance(km)	Means of Transportation	Carbon Emission Factor (kgCO₂e/km·kg)	Carbon Footprint (kgCO₂e)
Plastic parts	332.33 g	Dongguan, Shilong	Shenzhen, Longgang District	Highway	70.9	Truck	0.327	7.704
Silicone parts	22.4 g	Dongguan, Shilong	Shenzhen, Longgang District	Highway	70.9	Truck	0.327	0.519
Metal parts	73.08 g	Guangzhou, Yuexiu District	Shenzhen, Longgang District	Highway	139.7	Truck	0.327	3.334
Electrical parts	311.54 g	Dongguan, Shipai	Shenzhen, Longgang District	Highway	63.4	Truck	0.327	6.460
Packaging parts	622.9 g	Factory	Factory	Manual	0	–	–	–
Finished product	1362.25 g	Shenzhen, Longgang District	Wuhan	Railway	1036.2	Train	0.02782	39.2696

(4) Use stage

The power consumption of this Bluetooth speaker is 160 Wh; the service life is set at 2500 working cycles, with an average of two hours of use per cycle.

(5) Recycling and disposal stage

The recycling methods in the recycling stage mainly include shredding, incineration and landfilling, material recycling, and component recycling, among others. When recycling, the appropriate recycling method should be adopted for the components of the Bluetooth speaker based on actual conditions [32]. In the recycling and disposal stage of the STAGE Bluetooth speaker, some parts require more complex technical processing, such as batteries and capacitors, which are hazardous and need specialized treatment. These parts account for a small proportion, less than 3%. Therefore, this stage only calculates the materials with a large proportion in the STAGE Bluetooth speaker’s material list, such as plastics, aluminum, and paper materials, which account for 70%. Among these, the recycling conversion rate for plastics using density separation methods is approximately 98%, the highest conversion rate for aluminum is 98%, and the highest recycling rate for paper materials is 80% [33]. Professional electronic waste recycling, such as remelting ABS plastic and refining aluminum, applies a closed-loop benefit factor of 1.35 to the avoided emissions calculation. This accounts for cascading environmental benefits beyond direct material substitution, including reduced extraction of virgin resources and lower processing energy [31]. This coefficient comes from corporate data, and the carbon compensation coefficient is 3.31, which is determined by the characteristics of the recycled material: ABS plastic recycling emission reduction benefits: 16.6 kgCO₂e/kg × 98% recycling rate; aluminum closed-loop recycling benefit: 1.8 kgCO₂e/kg × 98% recycling rate. Implicit carbon amplification factor: 1.35. Calculation: (16.6 × 0.98 + 1.8 × 0.98)/ (16.6 + 1.8) × 1.35 = 3.31.

### Calculation results and analysis

Based on the LCA model, the total carbon footprint of the STAGE Bluetooth speaker over its life cycle was calculated as 227.01 kgCO₂e. The carbon emissions and percentage contributions of each stage are shown in Table 6.

This section provides a detailed breakdown of the carbon footprint calculations for each life cycle stage of the STAGE Bluetooth speaker [34], following the formulas defined in Table 1. All parameters are sourced from Tables 3–5 and referenced studies.

(1) Raw material acquisition Stage (Gm)

Formula: $\begin{array}{c} \text{Gm = }\sum {\mathrm{\backslash nolimits}}_{\text{i = 1}}^{\text{m}}\left({\text{M}}_{\text{i}}\times E{\text{F}}_{\text{i}}\right) \end{array}$

Parameters: ${\text{M}}_{\text{i}}$: Mass of material i (kg) – from Table 3; $\text{E}{\text{F}}_{\text{i}}$: Emission factor of material i (kgCO₂e/kg) – from Table 3.

Calculation: Key components (selected from Table 3):

ABS Main Frame:

GmABS = 0.14258 kg × 16.6 kgCO₂e/kg = 2.37 kgCO₂e

Aluminum Passive Radiator:

GmAluminum= 0.0612 kg × 1.8 kgCO₂e/kg = 0.11 kgCO₂e

Mainboard (Integrated Chips):

GmElectronics = 0.0415 kg × 29.33 kgCO₂e/kg = 1.22 kgCO₂e

Total Raw Material Emissions:

Gm = 2.37 kgCO₂e + 0.11 kgCO₂e + 1.22 kgCO₂e + (other materials) = 8.6 kgCO₂e

(2) Production and manufacturing stage (Gp)

Formula: $\text{Gp = }\sum {\mathrm{\backslash nolimits}}_{\text{i = 1}}^{\text{n}}\left({\text{E}}_{\text{i}}\times E{\text{F}}_{\text{i2}}\right)$

Parameters: ${\text{E}}_{\text{i}}$: Energy consumption of process i (kWh) – from Table 4; $\text{E}{\text{F}}_{\text{i2}}$: 0.413 kgCO₂e/kWh.

Calculation: Key components (selected from Table 4):

M11 (Soldering):

GpSoldering = 0.5 kWh × 0.413 kgCO₂e/kWh = 0.21 kgCO₂e

A5 (Speaker Assembly):

GpAssembly = 0.3 kWh × 0.413 kgCO₂e/kWh = 0.12 kgCO₂e

Total Production Emissions:

Gp = 0.21 kgCO₂e + 0.12 kgCO₂e + (other processes) = 4.13 kgCO₂e

(3) Transportation stage (Gt)

Formula: $\text{Gt = }\sum {\mathrm{\backslash nolimits}}_{\text{i = 1}}^{\text{n}}\left({\text{T}}_{\text{i}}\times {\text{D}}_{\text{i}}\times E{\text{F}}_{\text{i3}}\right)$

Parameters: ${\text{T}}_{\text{i}}$: Transported weight (kg) – from Table 5; ${\text{D}}_{\text{i}}$: Distance (km) manufacturer’s logistics data; $\text{E}{\text{F}}_{\text{i3}}$: Road = 0.327 kgCO₂e/km·kg, Rail = 0.02782 kgCO₂e/km·kg.

Calculation: Key components (selected from Table 5):

Plastic parts:

GtPlastic = 0.33233 kg × 70.9 km × 0.327 kgCO₂e/km·kg = 7.704 kgCO₂e

Silicone parts:

GtSilicone = 0.0224 kg × 70.9 km × 0.327 kgCO₂e/km·kg = 0.519 kgCO₂e

Metal parts:

GtMetal = 0.07308 kg × 139.7 km × 0.327 kgCO₂e/km·kg = 3.334 kgCO₂e

Electrical parts

GtElectrical = 0.31154 kg × 63.4 km × 0.327 kgCO₂e/km·kg = 6.460 kgCO₂e

Finished product:

GtFinished = 1.36225 kg × 1036.2 km × 0.02782 kgCO₂e/km·kg = 39.2696 kgCO₂e

Total Transportation Emissions:

Gt = 7.704 kgCO₂e + 0.519 kgCO₂e + 3.334 kgCO₂e + 6.460 kgCO₂e + 39.2696 kgCO₂e = 57.28 kgCO₂e

(4) Use stage (Gu)

Formula: $Gu=E\times {\text{T}}_{\text{w}}\times E{\text{F}}_{\text{i34}}\backslash )$

Parameters: E = 0.16 kWh/cycle (product specification); ${\text{T}}_{\text{w}}$ = 2500cycles (6–8 years lifespan); $\text{E}{\text{F}}_{\text{i34}}$ = 0.413 kgCO₂e/kWh (Southern China Power Grid).

Calculation: Gu = 0.16 kWh/cycle × 2500 cycles×0.413 kgCO₂e/kWh = 165.2 kgCO₂e

(5) Recycling and disposal stage (Gr)

Formula: $\begin{array}{c} \text{Gr = }\sum {\mathrm{\backslash nolimits}}_{\text{i = 1}}^{\text{n}}\left({\text{E}}_{\text{ir}}\times E{\text{F}}_{\text{i5}}\right)\text{- }\sum {\mathrm{\backslash nolimits}}_{\text{j = 1}}^{\text{n}}\left({\text{M}}_{\text{jr}}\times E{\text{F}}_{\text{jr}}\right) \end{array}$

Parameters: $\text{\textbackslash{} }{\text{E}}_{\text{ir}}$: Recycling energy (plastics: 0.5 kWh/kg); $\text{E}{\text{F}}_{\text{i5}}$ = 0.413 kgCO₂e/kWh; ${\text{M}}_{\text{jr}}$: Recycled ABS (0.14258 kg), Aluminum (0.0612 kg); $\text{E}{\text{F}}_{\text{jr}}$: Avoided emissions (ABS: 16.6 kgCO₂e/kg, Aluminum: 1.8 kgCO₂e/kg).

Calculation:

Recycling Energy Emissions:

$\sum {\mathrm{\backslash nolimits}}_{\text{i = 1}}^{\text{n}}\left({\text{E}}_{\text{ir}}\times E{\text{F}}_{\text{i5}}\right)\backslash )$ = [(0.14258 kg + 0.0612kg) × 0.5 kWh/kg ] × 0.413 kgCO₂e/kWh = 0.043 kgCO₂e

Avoided Emissions:

$\sum {\mathrm{\backslash nolimits}}_{\text{j = 1}}^{\text{n}}\left({\text{M}}_{\text{jr}}\times E{\text{F}}_{\text{jr}}\right)\backslash )$ = (0.14258 kg × 16.6 kgCO₂e/kg) + (0.0612 kg × 1.8 kgCO₂e/kg) = 2.477 kgCO₂e

Net Gr:

Gr = 0.043 kgCO₂e − (2.477 kgCO₂e × 3.31) = −8.2 kgCO₂e

Emissions in the use stage dominate, contributing 165.2 kgCO₂e, accounting for 72.77% of the total, mainly due to the long-term energy consumption during operation [6]. The transportation stage contributes 57.28 kgCO₂e (25.23%), as shown in Table 6.

**Table 6:** Carbon footprint calculation results of STAGE bluetooth speaker life cycle stages.

Stage	Raw Material Acquisition Stage	Production Stage	Transportation and Manufacturing Stage	Use Stage	Recycling and Disposa Stage	Total
Carbon Emission Total	8.6 kgCO₂e	4.13 kgCO₂e	57.28 kgCO₂e	165.2 kgCO₂e	−8.2 kgCO₂e	227.01 kgCO₂e
Percentage of Each Stage	3.78%	1.82%	25.23%	72.77%	−3.6%	100%

The full life cycle carbon footprint of the STAGE Bluetooth speaker calculated in this study (227.01 kgCO₂e) is notable for the dominance of the use stage, which is consistent with the trend of energy-intensive devices such as laptops [4]. The transportation stage value is significantly higher than typical values [35], primarily due to geographically dispersed suppliers and complex logistics networks. Raw material acquisition and production contribute 3.78% and 1.82%, equivalent to 8.6 kgCO₂e and 4.13 kgCO₂e respectively, while recycling yields a net reduction of −3.6%, corresponding to −8.2 kgCO₂e [36]. The dominance of the use stage is primarily driven by the product’s extended service life of 6–8 years and high energy consumption during operation. Transportation emissions are exacerbated by geographically dispersed suppliers and complex logistics networks [37].

### Sensitivity analysis

In order to verify the reliability of the traditional mass threshold (<1g) for carbon footprint results, this study sets up three scenarios for comparative analysis:

(1) Scenario A: Exclude all components < 1g (baseline).
(2) Scenario B: Include components < 1g with EF > 5 kgCO₂e/kg.
(3) Scenario C: Include all components < 1g.

As shown in Table 7, Scenario B includes high EF components, resulting in a negligible deviation of only +0.06%, while Scenario C includes all components below 1g, resulting in only a 0.10% increase in the total footprint. This confirms that omitting components below 1g is justified for this product, as their cumulative impact is minimal. It is worth noting that even high EF materials, such as alloy screws, do not contribute significantly due to their low mass fraction. These findings are consistent with Lee (2024), who reported that minor components with EF < 10 kgCO₂e/kg typically contribute less than 0.5% to the total footprint of electronic devices [11]. However, we need to be alert to the potential risks of trace components with high EFs, for example, special treatment may be required when the EF > 10 kgCO₂e/kg.

**Table 7:** Sensitivity analysis of minor component omission.

Scenario	Total Carbon Footprint (kgCO₂e)	Deviation
**A**	227.01	–
**B**	227.15	+0.06%
**C**	227.24	+0.10%

**Fig 5:**
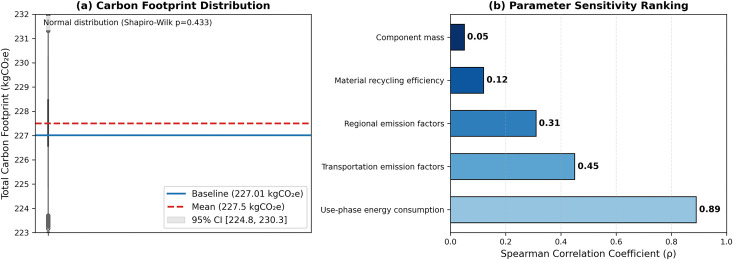
Monte carlo simulation results for carbon footprint uncertainty.

The Monte Carlo simulation (10,000 iterations) confirmed a normal distribution of the total carbon footprint (Shapiro-Wilk p = 0.433) with 95% confidence interval [224.8, 230.3] kgCO₂e, encompassing the baseline value of 227.01 kgCO₂e. See Fig 5a. Parameter sensitivity analysis identified use stage energy consumption (Spearman’s ρ = 0.89) and transportation EFs (ρ = 0.45) as the dominant uncertainty sources, while component mass (ρ = 0.05) and material recycling efficiency (ρ = 0.12) showed minimal impact. See Fig 5b.

## Discussion

The results of this study are consistent with existing research results on carbon footprint assessment of consumer electronics products and fill the gaps in previous literature through methodological innovation. Through comparison with existing research and in-depth analysis of influencing factors, this paper further clarifies the optimization path of low-carbon design of Bluetooth speakers and its academic value. Consistent with Cordella (2021) study on smartphones [6], the dominance of carbon emissions in the use stage of Bluetooth speakers (72.77%) reveals a common challenge for electronic products: the cumulative effect of long-term energy consumption on carbon footprint. However, the significant contribution of the transportation stage (25.23%) highlights the product’s specificity, the geographical dispersion of the spare parts supply chain and the reliance on road transportation. This phenomenon is common in the electronic product manufacturing of enterprises, which echoes the research conclusions of Chiang and Che (2015) on logistics efficiency [38]. Further analysis found that although ABS plastic only accounts for 14.3% of the mass, it contributes 28% of raw material emissions. Its high EF confirms Kuo (2014) criticism of the environmental cost of polymer materials, indicating that lightweight design needs to be promoted in conjunction with material substitution [39]. As visualized in Fig 5, the Monte Carlo simulation confirmed a normal distribution of carbon footprint (Shapiro-Wilk p = 0.433) with 95% confidence interval [224.8, 230.3] kgCO₂e. The sensitivity ranking identifies use stage energy consumption (ρ = 0.89) and transportation EFs (ρ = 0.45) as dominant uncertainty sources, guiding priority mitigation strategies.

Based on the analysis, this study proposes the full life cycle of the above path:

(1) Optimize energy consumption in the use stage: reducing single-cycle power consumption by 10% through circuit design improvements can reduce 16.5 kgCO₂e emissions; combining renewable energy power supply, such as solar charging, can further alleviate the impact of long-term energy consumption.
(2) Reconstructing supply chain and logistics: supplier localization, transportation route optimization and lightweight design, such as using bio-based plastics instead of ABS, can reduce transportation emissions by more than 20%.
(3) Material substitution and recycling: using bio-based plastics or recycled aluminum can reduce raw material emissions by 50%, while indirectly reducing transportation emissions by reducing weight; improving the recycling efficiency of plastic and paper materials can expand the carbon offset effect.

The innovation of the research method is reflected in two aspects: first, by integrating enterprise production data with GaBi regional database parameters, the error of the hybrid LCA is controlled at 0.10%, solving the problem of data fragmentation in enterprises. Its accuracy is comparable to that of the tool PIQET [12]; second, while verifying the reliability of the traditional 1g mass threshold, a correction principle for the mandatory inclusion of high-EF trace materials is proposed, responding to the concerns of Lee (2024) about trace components [11]. However, the failure to include CH₄/ N₂O and the limitations of battery recycling may underestimate the true emissions, and in the future, it will be necessary to combine the dynamic EF model to expand the system boundaries [16]. In summary, this study not only verifies the applicability of the LCA method in specific products but also provides the industry with a systematic reference framework from data integration to design optimization.

## Conclusion

The carbon footprint of the STAGE Bluetooth speaker over its entire life cycle is 227.01 kgCO₂e, of which the use stage (72.77%) and the transportation stage (25.23%) are the key emission sources, driven by long-term product energy consumption and geographical dispersion of the supply chain. By integrating enterprise data and regionalized parameters, this study constructed a carbon accounting model suitable for enterprises, verified the applicability of the 1g mass threshold, and proposed correction principles for trace materials with high EFs.

In practice, four strategies have significant emission reduction potential:

(1) A 10% reduction in energy consumption during the use stage can reduce 16.5 kgCO₂e;
(2) Localization of suppliers and optimization of transportation modes can reduce transportation emissions by more than 20%;
(3) Replacing ABS with bio-based plastics can reduce raw material emissions by 50%;
(4) Recycling processes driven by renewable energy can improve carbon offset efficiency.

Future research needs to expand the scope of CH₄/ N₂O accounting, develop a dynamic supply chain emission model, and establish a carbon data platform through industry collaboration to provide systematic support for the low-carbon transformation of consumer electronics products. This study provides a scientific framework for the low-carbon design of Bluetooth speakers and establishes a methodological foundation for carbon management in the electronics industry.

## Supporting information

S1 Fig STAGE Bluetooth speaker structure display. (DOCX)

S2 Fig Establishment of a quantitative assessment model for carbon emissions of Bluetooth speakers at this stage. (DOCX)

S1 Data Bluetooth speaker parts information collection. (DOCX)

S2 Data List of the STAGE Bluetooth speaker materials and carbon emission factor. (DOCX)

S3 Data Energy consumption of STAGE Bluetooth speaker during production. (DOCX)

S4 Data Data of the main transportation stages of STAGE Bluetooth speakers. (DOCX)
