# Supplementary figures and images for: Developing a carbon footprint calculation method for product life cycle based on low-carbon design: A case study of the STAGE Bluetooth speaker
Source: PLoS One. 2025 Aug 20;20(8):e0327576. doi: 10.1371/journal.pone.0327576 (PMC12367186; doi:10.1371/journal.pone.0327576)

S1 Fig. STAGE Bluetooth speaker structure display. (DOCX)


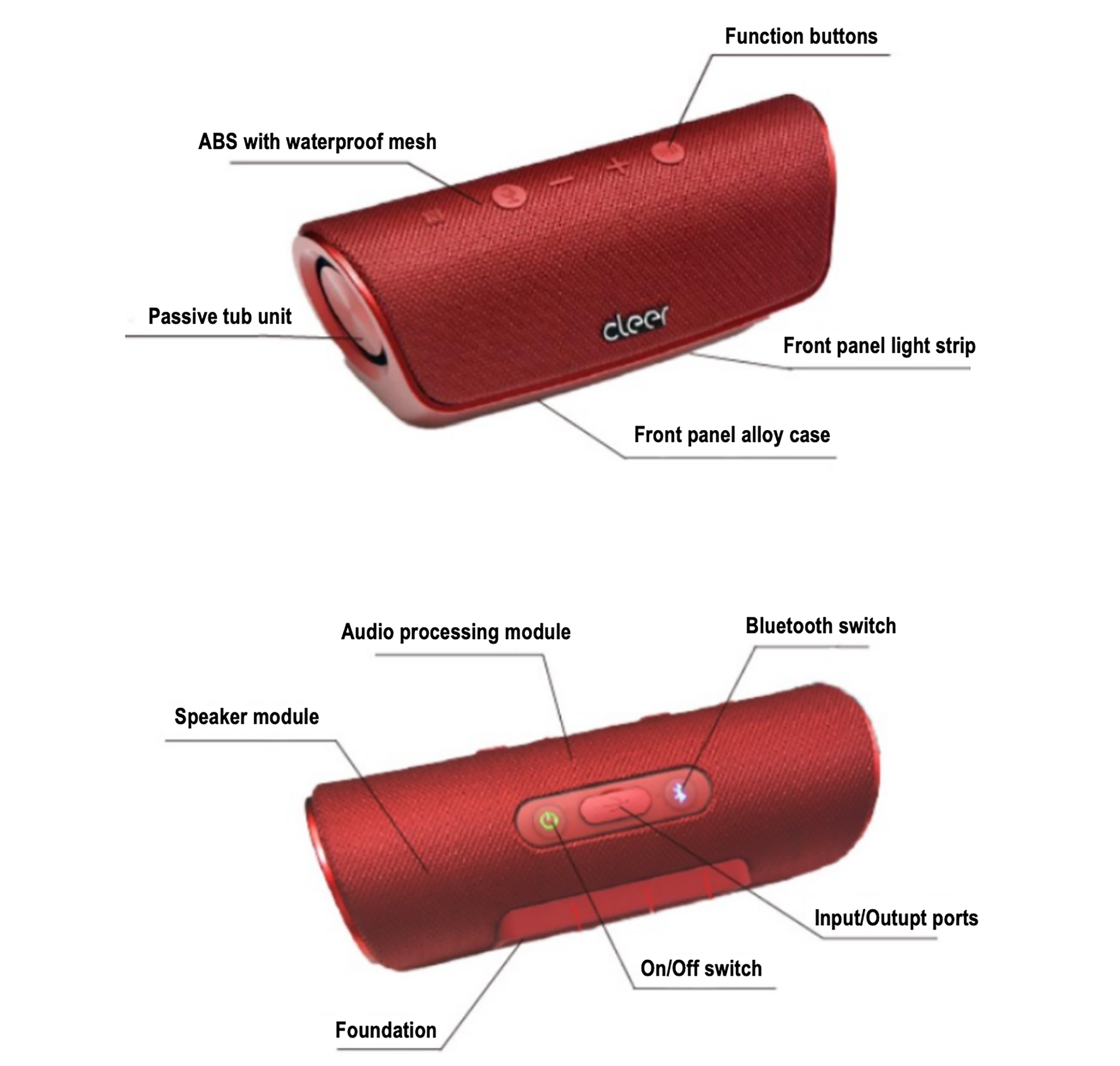


**Fig. STAGE Bluetooth Speaker Diagram**

Supplement: S1 Fig — (DOCX) [file pone.0327576.s001.docx]
