# Supplementary material for: Developing a carbon footprint calculation method for product life cycle based on low-carbon design: A case study of the STAGE Bluetooth speaker
Source: PLoS One. 2025 Aug 20;20(8):e0327576. doi: 10.1371/journal.pone.0327576 (PMC12367186; doi:10.1371/journal.pone.0327576)
Supplement: S2 Fig — (DOCX) [file pone.0327576.s002.docx]

S2 Fig. Establishment of a quantitative assessment model for carbon emissions of Bluetooth speakers at this stage. (DOCX)


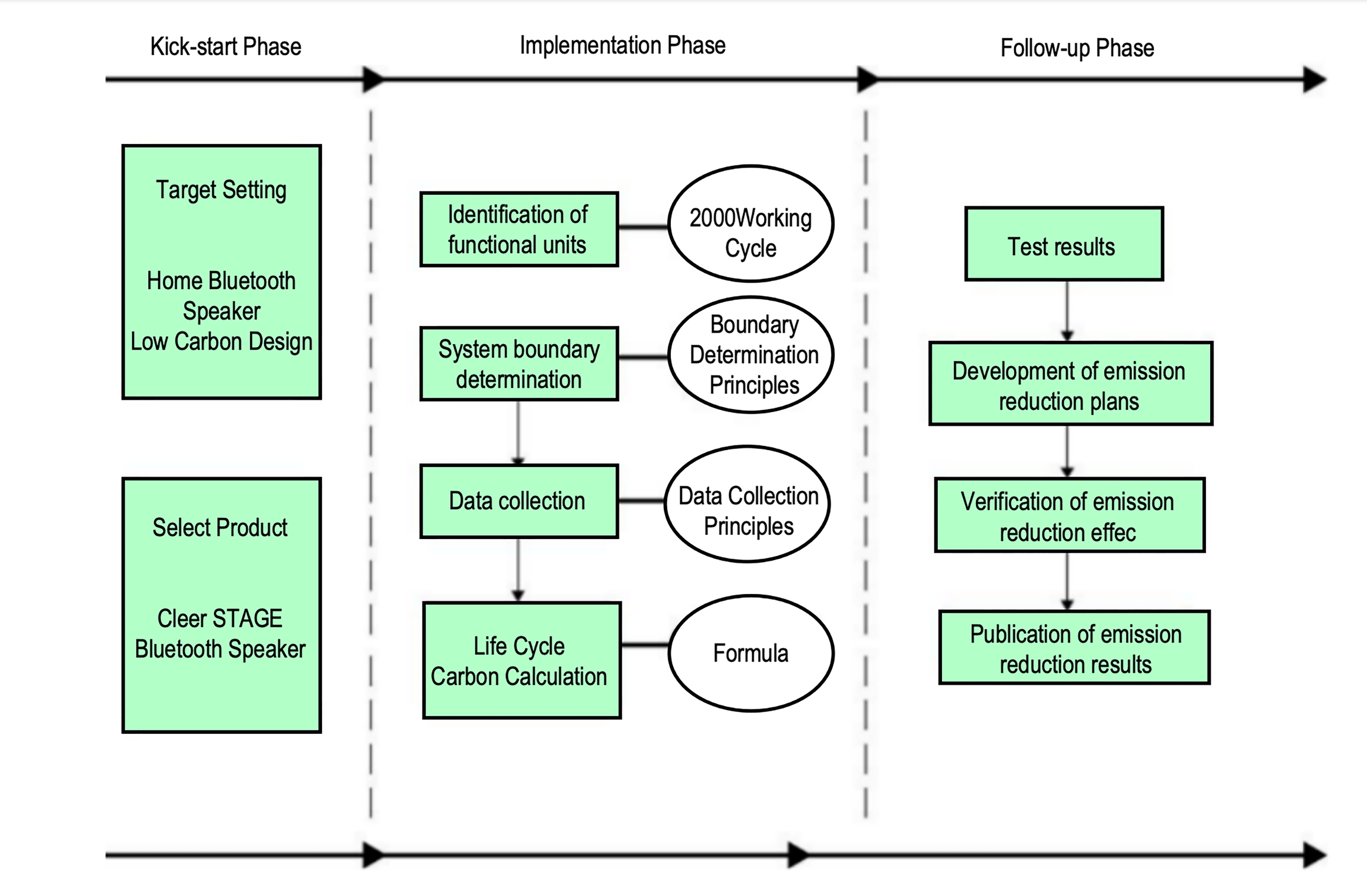


**Fig. STAGE Bluetooth Speaker Carbon Emission Quantitative Assessment Model**
