# Supplementary material for: Developing a carbon footprint calculation method for product life cycle based on low-carbon design: A case study of the STAGE Bluetooth speaker
Source: PLoS One. 2025 Aug 20;20(8):e0327576. doi: 10.1371/journal.pone.0327576 (PMC12367186; doi:10.1371/journal.pone.0327576)
Supplement: S1 Data — (DOCX) [file pone.0327576.s003.docx]

S1 Dataset. Bluetooth speaker parts information collection. (DOCX)

| **Pseudolaric acid** | | **Cleer Bluetooth Speaker** |  |
| --- | --- | --- | --- |
| **Product Model** | | **STAGE** |  |
| **Structural description** | | | |
| **System** | **Component Name** | **Detailed Description** | |
| **Shell and Bracket System** | Main Frame | The inner structure frame of the speaker | |
|  | Decorative Strip | ABS material decorative strip, installed outside the frame, serves to cover and protect the interior and contributes to the overall aesthetic | |
|  | Bottom Non-slip Pad | Increases the stability of the speaker placement | |
|  | Speaker Net | Speaker net component is composed of ABS frames with attached fabric on the outside; functions to protect the speaker and prevent dust | |
|  | Metal Radiating Diaphragm Unit | Oscillates with the rhythm of the music | |
| **Electronic Component System** | MAIN Mainboard Component | Main control board | |
|  | USB & AUX Board Component | Output/input unit | |
|  | ON & OFF Board Component | Switch | |
|  | LED Board Component | LED lights | |
|  | KEY Board Component | Development board | |
|  | Speaker L48 | Speaker unit | |
| **Power Supply System** | Battery Group | Cylindrical lithium-ion battery (7.4V, 2600mAh) | |
|  |  |  | |
| **Packaging System** | User manual, EVA shockproof, color box, inner support, outer box, stickers | Packaging for logistics and product sales | |

**STAGE Bluetooth Speaker Parts Information**
