# Supplementary material for: Developing a carbon footprint calculation method for product life cycle based on low-carbon design: A case study of the STAGE Bluetooth speaker
Source: PLoS One. 2025 Aug 20;20(8):e0327576. doi: 10.1371/journal.pone.0327576 (PMC12367186; doi:10.1371/journal.pone.0327576)
Supplement: S2 Data — (DOCX) [file pone.0327576.s004.docx]

S2 Dataset. List of the STAGE Bluetooth speaker materials and carbon emission factor (DOCX)

| **Material type** | **Component Name** | **Material** | **Quantity** | **Total Weight (g)** | **Carbon Emission Factor (kgCO₂e)** | **Carbon Footprint (kgCO₂e)** |
| --- | --- | --- | --- | --- | --- | --- |
| **plastic part** | Main Frame | ABS | 1 | 142.58 | 16.6 | 2.367 |
|  | Function Key Cover | ABS | 1 | 4.6 | 16.6 | 0.076 |
|  | Diaphragm Bracket | ABS | 2 | 34.2 | 16.6 | 0.57 |
|  | Main Foot Stand | ABS | 1 | 13.5 | 16.6 | 0.22 |
|  | LED Back Cover | ABS | 1 | 5.28 | 16.6 | 0.088 |
|  | Decorative Strip | PC | 1 | 31.6 | 0.2 | 0.006 |
|  | Function Key Top Cover | PC | 1 | 4.1 | 0.2 | 0.0008 |
|  | USB Cover | TPU | 1 | 1.25 | 4.25 | 0.018 |
|  | TYPEC Input Box Top Cover | TPU | 1 | 4.65 | 4.25 | 0.02 |
|  | Speaker Grid Component | ABS Component/Waterproof Cloth | 1 | 90.5 | 16.6 | 1.5 |
| **Silicone parts** | Main Bracket Rubber | Rubber | 1 | 17.7 | 2.4 | 0.29 |
|  | Sealing Ring | Rubber | 2 | 4.7 | 2.4 | 0.0113 |
| **hardware** | Screw | Carbon Steel | 58 | 11.88 | 3.2 | 0.038 |
|  | Passive Basin | Aluminum | 2 | 61.2 | 1.8 | 0.11 |
| **electronic component** | PVC Line | PVC | 2 | 3.6 | 7 | 0.025 |
|  | MAIN Mainboard Component (Integrated Chip) | Component | 1 | 41.5 | 29.33 | 1.22 |
|  | USB & AUX Board Component | Component | 1 | 1.95 | 29.33 | 0.06 |
|  | ON & OFF Board Component | Component | 1 | 1.93 | 29.33 | 0.06 |
|  | LED Board Component | Component | 1 | 2 | 29.33 | 0.059 |
|  | KEY Board Component | Component | 1 | 3.06 | 29.33 | 0.09 |
|  | Speaker L48 | Component | 2 | 140 | 29.33 | 0.533 |
| **other** | Battery Group | Component | 1 | 99 | 6.31 | 0.624 |
|  | Sticker | Copper Plate Paper | 11 | 1.1 | 3 | 0.0033 |
|  | Color Box (including inner holder) | Gray Card Paper | 1 | 403 | 1.7 | 0.68 |
|  | Instruction Manual | Copper Plate Paper | 1 | 6.8 | 3 | 0.0204 |
|  | Shockproof EVA | EVA | 16 | 4.6 | 2.82 | 0.013 |

**STAGE Bluetooth Speaker Bill of Materials and Carbon Emission Factors**
