# Supplementary material for: Developing a carbon footprint calculation method for product life cycle based on low-carbon design: A case study of the STAGE Bluetooth speaker
Source: PLoS One. 2025 Aug 20;20(8):e0327576. doi: 10.1371/journal.pone.0327576 (PMC12367186; doi:10.1371/journal.pone.0327576)
Supplement: S3 Data — (DOCX) [file pone.0327576.s005.docx]

S3 Dataset. Energy consumption of STAGE Bluetooth speaker during production (DOCX)

| **#** | **Manufacturing Step** | **Labor** | **Energy Source (Electricity/Steam/Natural Gas/Oil/Biofuel, etc.)** |
| --- | --- | --- | --- |
| **T1** | Mainboard Programming | 1 | Electricity |
| **T2** | Mainboard UI Testing | 1 | Electricity |
| **M1** | Mainboard High-temperature Glue and Conductive Cloth Application | 1 | Electricity |
| **M2** | Mainboard and Battery Foam Wrapping | 1 | Manual Work |
| **M3** | Battery Bracket Installation and Fastening on Mainboard | 1 | Manual Work |
| **M4** | Microphone Component Assembly and Installation into Microphone Box Lower Cover | 1 | Manual Work |
| **M5** | Gluing of Microphone Box Lower Cover Slot | 1 | Manual Work |
| **M6** | Upper Cover Installation and Screw Fixation for Microphone Box | 1 | Electricity |
| **M7** | Microphone Component Waterproofing and Foam Installation | 1 | Manual Work |
| **M8** | Function Key Cable Routing and Plug into Key Panel | 1 | Manual Work |
| **M9** | Function Key Upper/Lower Cover and Button Panel Screw Fixation | 1 | Electricity |
| **M10** | Input `Box Upper Cover Laser Engraving | 1 | Electricity |
| **M11** | USB Board and ON/OFF Board Soldering | 1 | Electricity |
| **M12** | Input Box Upper Cover Assembly: Key Bracket Installation and USB Board Foam Sticking | 1 | Manual Work |
| **M13** | USB Board Cable Routing, Installation into Input Box Lower Cover and Gluing | 1 | Manual Work |
| **M14** | USB Cover Installation into Upper Cover, Screw Fixation and Gluing | 1 | Manual Work |
| **M15** | LED Panel Cable Assembly, Installation into Light Guide and Waterproofing | 1 | Manual Work |
| **M16** | LED Back Cover Screw Fixation and Waterproofing | 1 | Manual Work |
| **M17** | Main Body Air Vents and Double-sided Tape Application, Microphone FPC Foam Wrapping | 1 | Manual Work |
| **M18** | Single Speaker Testing | 1 | Electricity |
| **M19** | Speaker Wire Soldering | 1 | Electricity |
| **M20** | Speaker Wire Vibration Cushion and Speaker Foam Application | 1 | Manual Work |
| **A1** | Light Guide Installation onto Main Body, FPC Foam Wrapping | 1 | Manual Work |
| **A2** | Button Component and Microphone Component Adhesion onto Main Body, FPC Foam Wrapping | 1 | Manual Work |
| **A3** | Input Box Component Adhesion onto Main Body, FPC Foam Wrapping | 1 | Manual Work |
| **A4** | Mainboard Installation, Cable Plugging, Foam Application | 1 | Manual Work |
| **A5** | Main Body Silicone Seal Installation, Speaker Socket Assembly | 1 | Electricity |
| **A6** | Semi-finished Product Functional Testing | 1 | Electricity |
| **A7** | Speaker Installation and Screw Fixation | 1 | Electricity |
| **A8** | Left and Right Vibration Plate Bracket Seal Ring Installation | 1 | Manual Work |
| **A9** | Left and Right Vibration Plate Bracket Installation onto Main Body | 1 | Manual Work |
| **A10** | Bracket Gluing, Passive Disc Screw Fixation | 1 | Electricity |
| **A11** | Power Testing | 1 | Electricity |
| **A12** | All Cable Port and Speaker Area Sealing | 1 | Manual Work |
| **A13** | Waterproof Testing | 1 | Electricity |
| **A14** | Decorative Strip Installation into Main Body and Screw Fixation | 1 | Electricity |
| **A15** | Speaker Mesh Fastening into Main Body and High-temperature Tape Wrapping | 1 | Electricity |
| **A16** | Sweep Frequency Testing | 1 | Electricity |
| **A17** | Phone Function Testing | 1 | Electricity |
| **A18** | Electroacoustic Testing | 1 | Electricity |
| **A19** | Product Charging | 1 | Electricity |
| **A20** | Appearance Check | 1 | Manual Work |

**Energy Consumption of STAGE Bluetooth Speaker During Production**
