# Supplementary material for: Developing a carbon footprint calculation method for product life cycle based on low-carbon design: A case study of the STAGE Bluetooth speaker
Source: PLoS One. 2025 Aug 20;20(8):e0327576. doi: 10.1371/journal.pone.0327576 (PMC12367186; doi:10.1371/journal.pone.0327576)
Supplement: S4 Data — (DOCX) [file pone.0327576.s006.docx]

S4 Dataset. Data of the main transportation stages of STAGE Bluetooth speakers (DOCX)

| Item | Transportation Weight | Origin | Destination | Mode | Distance（km） | Means of Transportation | Carbon Emission Factor (kgCO₂e/km·kg） | Carbon Footprint (kgCO₂e) |
| --- | --- | --- | --- | --- | --- | --- | --- | --- |
| Plastic parts | 332.33g | Dongguan, Shilong | Shenzhen, Longgang District | Highway | 70.9 | Truck | 0.327 | 7.704 |
| Silicone parts | 22.4g | Dongguan, Shilong | Shenzhen, Longgang District | Highway | 70.9 | Truck | 0.327 | 0.519 |
| Metal parts | 73.08g | Guangzhou, Yuexiu District | Shenzhen, Longgang District | Highway | 139.7 | Truck | 0.327 | 3.334 |
| Electrical parts | 311.54g | Dongguan, Shipai | Shenzhen, Longgang District | Highway | 63.4 | Truck | 0.327 | 6.460 |
| Packaging parts | 622.9g | Factory | Factory | Manual | 0 | - | - | - |
| Finished product | 1362.25g | Shenzhen, Longgang District | Wuhan | Railway | 1036.2 | Train | 0.02782 | 39.2696 |

**Data List of Main Transportation Stages of STAGE Bluetooth Speaker**
